# Compositionality in the language of emotion

**DOI:** 10.1371/journal.pone.0201970

**Published:** 2018-08-15

**Authors:** Federica Cavicchio, Svetlana Dachkovsky, Livnat Leemor, Simone Shamay-Tsoory, Wendy Sandler

**Affiliations:** 1 Sign Language Research Lab, University of Haifa, Haifa, Israel; 2 Department of Psychology, University of Haifa, Haifa, Israel; Universita degli Studi di Udine, ITALY

## Abstract

Emotions are signaled by complex arrays of face and body actions. The main point of contention in contemporary treatments is whether these arrays are discrete, holistic constellations reflecting emotion categories, or whether they are compositional—comprised of smaller components, each of which contributes some aspect of emotion to the complex whole. We address this question by investigating spontaneous face and body displays of athletes and place it in the wider context of human communicative signals and, in particular, of language. A defining property of human language is compositionality—the ability to combine and recombine a relatively small number of elements to create a vast number of complex meaningful expressions, and to interpret them. We ask whether this property of language can be discerned in a more ancient communicative system: intense emotional displays. In an experiment, participants interpreted a range of emotions and their strengths in pictures of athletes who had just won or lost a competition. By matching participants’ judgements with minutely coded features of face and body, we find evidence for compositionality. The distribution of participants’ responses indicates that most of the athletes’ face and body features contribute to displays of dominance or submission. More particular emotional components related, for example, to positive valence (e.g. happy) or goal obstruction (e.g. frustrated), were also found to significantly correlate with certain face and body features. We propose that the combination of features linked to broader components (i.e, dominant or submissive) and to more particular emotions (e.g, happy or frustrated) reflects more complex emotional states. In sum, we find that the corporeal expression of intense, unfiltered emotion has compositional properties, potentially providing an ancient scaffolding upon which, millions of years later, the abstract and constrained compositional system of human language could build.

## Introduction

There are two main opposing scientific approaches to the relation between the expression of an emotion and its interpretation: discrete—here called **holistic**—and **compositional**. We begin by describing the two approaches, which have been supported primarily by research on facial expression. Our own approach includes both face and body, and the introductory exposition continues to consider the role of the body in displays of emotion. We motivate the present study by considering compositionality more closely, and explaining how the concept, known to be fundamental to human language, is understood in the context of intense emotion. We then proceed to our study. Our investigation uses pictures of athletes who have just won or lost a sports competition to seek how participant judgements of nine emotions/emotional states and maps onto different configurations of face and body. As we will show, the study supports a compositional interpretation of face and body configurations in the expression of intense emotion, suggesting that human communication in emotion as in language is quintessentially compositional.

### Holistic and compositional approaches to the study of emotion

According to the holistic approach, a small number of emotions considered to be basic are hard-wired (genetically determined) in the brain, and are always linked to specific facial expressions, both in production and in recognition [1]. Ekman and colleagues have spurred a large body of research on facial expression showing that a set of six basic emotions are reliably and universally identified from particular configurations of facial actions [2–4]. All other facial expressions can be regarded as the result of mixtures (blends) of basic emotions. However, among the challenges to this holistic view is the discovery that, in naturalistic data, the proposed basic facial expressions rarely actually occur, even where expected [5]. Another problem for the holistic approach is that, although some basic emotions such as fear and surprise share several facial components, there is no way to account for these commonalities if we adhere to the postulate that facial expressions of basic emotions are discrete gestalts.

Therefore, several researchers have argued that it is necessary to go beyond holistic (discrete) emotions. An opposing school of research has sought to demonstrate that emotional displays are composites of signals conveying different components of affective meaning. This latter view, put forward in work by Smith, Russell, Scherer, and others [6–14], is conceptually compatible with the approach we propose here, and we call it the compositional approach. This broad framework is represented by several directions of research. For example, according to the **dimensional** approach, affective states are not independent of one another; rather, they are related to one another in a systematic manner. In this approach, most affect variability is subsumed under three dimensions: Valence, Arousal, and Potency (Dominance) [15]. The Valence dimension refers to how Positive or Negative an emotion is, and ranges from unpleasant to pleasant feelings. The Arousal dimension refers to how excited or apathetic the emotion is, and it ranges from sleepiness or boredom to frantic excitement. The Dominance (potency) dimension refers to the degree of power expressed. Note, however, that the dimensional approach does not claim that these three dimensions are the only dimensions sufficient to differentiate between all emotions.

Scherer and colleagues extended the dimensional approach by introducing the componential model of emotion, which is based on their appraisal theory [16]. The appraisal-based approach focuses on the complex mechanisms generating emotion and posits that emotions are generated through continuous, recursive, subjective evaluation of different components related to our own internal state and the state of the outside world. Emotion is described through a set of stimulus evaluation checks, such as novelty, intrinsic pleasantness, goal-based significance, and coping potential (For further details on different approaches to human emotions and their expressions, see Scherer [17] and Grandjean et al. [18]).

### Emotion and the body

The holistic emotion approach that has been a prevalent scientific paradigm for several decades traditionally focused primarily on facial expression, and the expressive information in body movements has been under-investigated in the field of emotion research. Yet, once compositional approaches entered the scientific stage, exclusive focus on the face began to be questioned [19, 20]. Russell [21] found that participants rating faces could only assess the level of arousal and whether the expressed feelings were Positive or Negative, but could not interpret specific emotions. Interestingly, only when the context was known could specific emotions be recognized from the face. He also claims in his work that the face does not convey more information about emotions than the rest of the body (e.g. body posture, as well as words and intonation), but rather signals the global feelings of a person. Scherer and colleagues have shown that body postures convey important emotional information [22–24] and De Gelder has conducted experiments that map different body postures to the expression of particular emotions [25–27].

A series of studies by Aviezer and colleagues considered both face and body in the investigation of the expression of intense emotion. In a study involving pictures of tennis players who had just won or lost a point, the researchers [28] interpreted win and loss in terms of Valence, making the a priori assumption that winners express Positive emotions and losers Negative ones. For participants in their experiment, the body was a better indicator of Positive vs. Negative emotion than was the face. The authors concluded that facial expressions in these displays are not a reliable source of Valence information, whereas body postures are. They followed up this study with one that used stimuli posed by actors, and targeted facial expression specifically, finding that participants could not accurately identify Positive faces of the athletes [29].

The current study also focuses on intense emotion in athletes, but is different from Aviezer’s work in its assumptions, methodology, and results, as we explain in the rest of this paper. We place the expression of intense emotion in the broader context of human communication, which also includes language. Language arrived quite recently on the evolutionary scene, a mere 40,000–100,000 years ago, while emotional expression is surely much, much older, judging from the behaviors of related species, such as chimpanzees, who share a common ancestor with humans [30]. Our point of entry is **compositionality**, a central organizing principle of language. If we find that emotional expression has compositional characteristics, we would be taking a step toward understanding fundamental underpinnings of human communication that eventually led to language.

#### Compositionality

Compositionality refers to the ability to create and interpret complex messages by selecting and recombining component parts, where each is associated with its own meaning and contributes to the interpretation of the whole. This property pervades language at every level in a hierarchy, from combinations of sounds that make words, to words that make phrases, phrases that make sentences, and sentences that combine to make more complex sentences. For example, recombining three sounds can give three different words: *act*, *tac*, *cat*.

A distinction is often made between combinatoriality, in which units such as sounds do not have to be meaningful in order to contribute to different meanings, and compositionality, in which the units are necessarily meaningful (e.g, de Boer et al. [31]). Others characterize both types as combinatorial [32,33]. As we seek to determine whether the units we identify are ‘meaningful’ in the sense that they correspond to aspects of emotion, we choose to use the term *compositional* in our inquiry.

Moving up the language hierarchy, combining meaningful word parts such as *treat*, *move*, *-able*, *un-* gives us the complex words, *treatable*, *movable*, *untreatable*, *unmovable*. *Cat*, *small*, *the* or *mouse*, *scrawny*, *devoured* combine according to rules to give us the phrases *the small cat*, *devoured the scrawny mouse*. Phrases combine according to rule to make sentences: *The small cat devoured the scrawny mouse*. Going one step further, sentences can be more complex by conjoining them (*The small cat devoured the scrawny mouse and took a nap*) or embedding one inside another (*The small cat that devoured the scrawny mouse took a nap*). Each level consists of components with their own meanings, and builds up complexity by combining them.

Compositionality pervades not only spoken languages, but also **sign languages**, natural languages which arise spontaneously in deaf communities and which are structured like spoken languages in many important ways [34]. The important difference is that they are conveyed entirely in the visual domain and exploit nearly every visible part of the body to do so. Meaningful components of an utterance can be articulated simultaneously by different parts of the body to convey a great deal of complexity. This observation guides our investigation of emotional displays by facial and bodily articulators. For rationale and overview of this approach see [35].

Thus, the existence of a compositional system, in which spontaneous complex expressions are comprised of simpler ones which distinguish one from another and contribute their interpretations to the whole, is fundamental to human communication in terms of its complexity and versatility. Here we ask whether compositionality is the quintessential principle of human communication more broadly, starting from involuntary expressions of emotion. Our study considers the merits and limitations of previous work by using spontaneous and natural stimuli. In addition, the compositional approach which we adopt here accounts for some of the unexplained discrepancies in the relative contributions of face and body. At the outset, we offer a caveat. We do not assume that emotional expressions manifest the same degree of complexity, hierarchy, strict combinatorial rules, or context independence as language. Emotion is not language. However, we introduce the question of whether the fundamental property of language, compositional internal structure, characterizes this ancient communicative system as well, and might have provided a foundation for the later evolution of language.

In an experiment that aims to tackle these questions, participants interpreted a range of emotions and their strengths in pictures of athletes who had just won or lost a competition. We hypothesized that their judgments would rely on compositional properties of emotion displays, where individual components of face and body contribute some aspect of meaning to the complex whole. More specifically, we hypothesize that if emotions are interpreted compositionally, individual components of face and body can be reliably associated in their distribution with a specific interpretation, and may recombine, lending to different emotion arrays. The opposite, holistic approach, would predict that interpreting the meaning of an emotion requires conglomerates of features that cannot be individually interpreted reliably. Therefore, co-occurrence of the individual components will be fixed, and each emotion will only be identifiable as gestalts.

## Method

### Participants

We recruited 84 right handed participants (18 to 43 years, mean age 24.77), 64 females and 20 males, all graduate students at the University of Haifa and the Technion. The gender imbalance resulted from a similar imbalance in student enrollment in departments from which participants were recruited and is not expected to affect results. Although some authors have suggested a gender difference in the expression [36] and perception of facial expression of emotions [37], Cavicchio and Sandler [38] found that in naturalistic extreme emotion displays men and women do not differ in their facial and body expressions. Furthermore, regarding emotion perception, a recent study conducted on 6102 participants showed that, overall, there is no gender difference in the perception of emotions [39].

All participants had normal or corrected vision and provided written informed consent to participate in this. The study was approved by the Research authority at the University of Haifa ().

### Stimuli

Many previous studies have shown that acted emotions are perceived differently than real emotions [40–42]. These findings reveal limitations of studies that use actors and posed emotions, in emotion research, if the goal is to study genuine felt emotions. In the present study, we try to overcome this limitation by capturing spontaneous facial expressions and body postures of athletes' pictures taken moments after they have won or lost a high-stakes competition. We selected 143 pictures from an earlier study by Cavicchio and Sandler [38]. In that study, 350 pictures of athletes were coded for features of facial expression and body posture. The facial expressions portrayed in the pictures were coded using the Facial Action Coding System (FACS), [43]. All pictures were coded by a certified and experienced FACS coder. A second certified FACS coder checked 60 randomly chosen pictures. Coders reached a high inter coder reliability (kappa = 0.79, p<0.01). The body was coded using the Body Arrangement Coding System that our team of coders devised, validated and checked for reliability [44]. Using a Multiple Component Analysis procedure, Cavicchio and Sandler determined which features correlated significantly with victory and which with loss, providing a bench mark for prototypical win and loss displays as a basis for the present study. Winning athletes typically produced a more complex set of facial expressions than losing athletes. For upper face, AUs 4 (brow lowerer), 6 (cheek raiser) and 7 (lid tightener) in combination were highly correlated with win. For lower face, AUs 12 (lip corners up), 20 and 27 (lip and mouth stretch) were highly correlated with win. In contrast, loss was typically characterized by neutral or “not visible” facial features. As regards the body, winners’ bodies are open and extended while those of losers are closed, often kneeling on the ground, and appear diminished in size.

**Fig 1:**
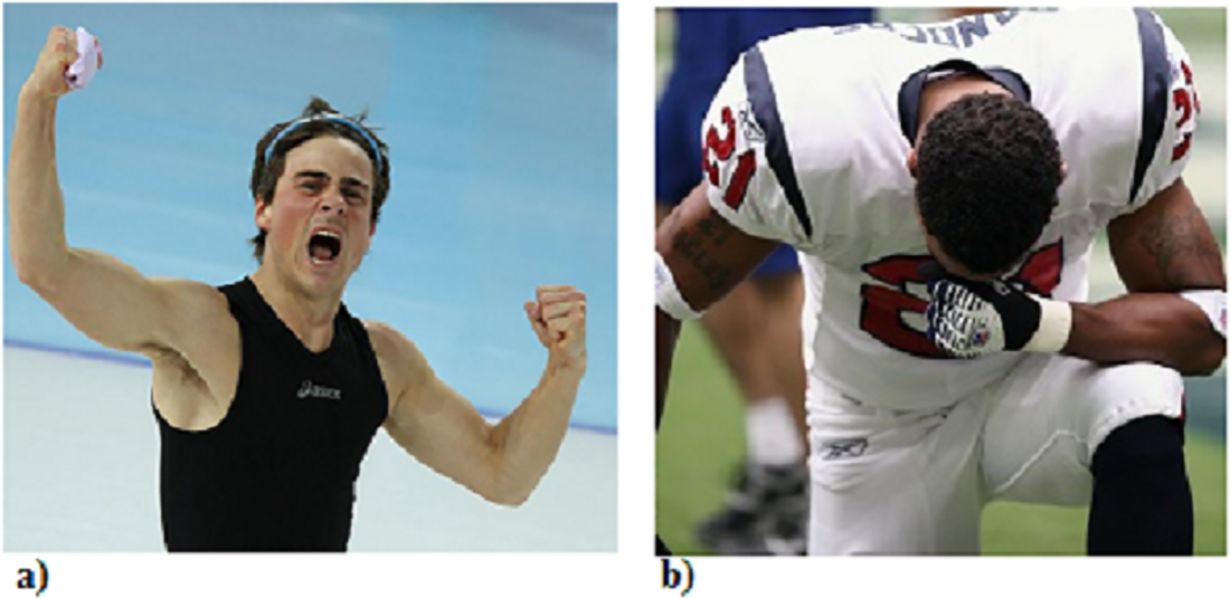
(a) Prototypical victory display; (b) prototypical defeat display. Pictures are licensed under CC by 4.0. Credits go respectively to M. Smelter and A. Gockowski.

**Fig 2:**
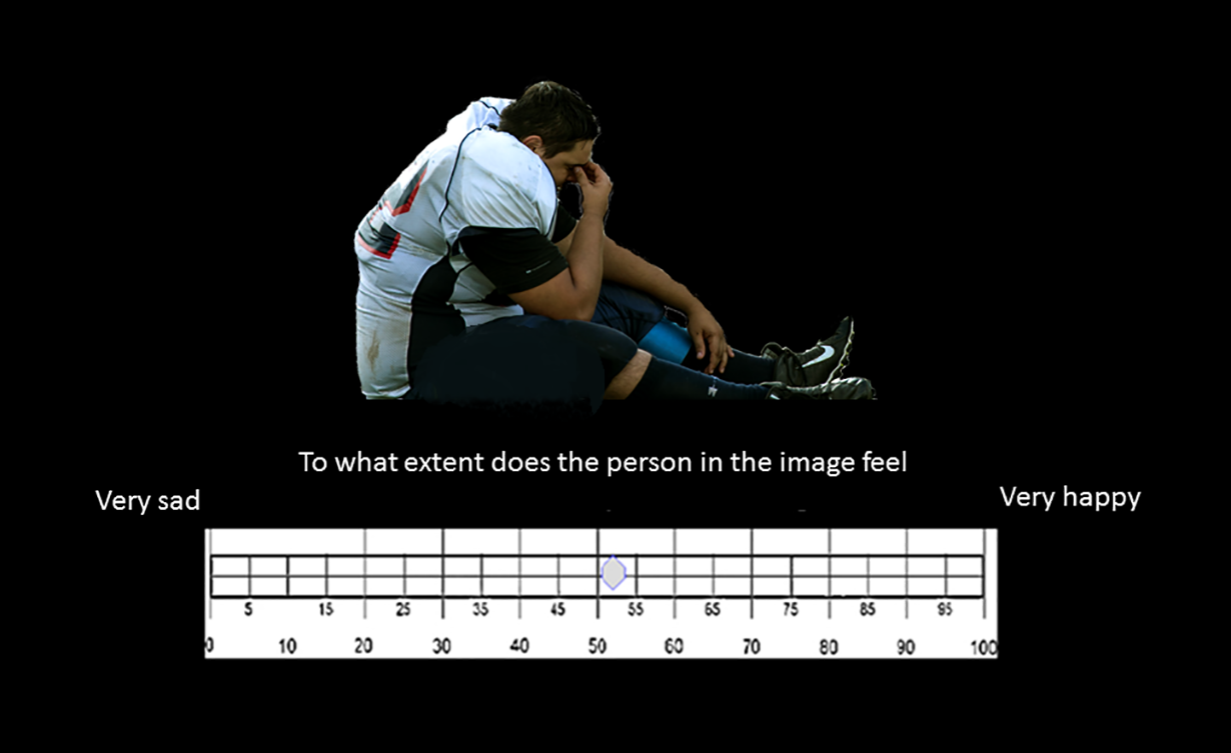
Example of the experimental procedure, with sample picture of an athlete, and a scale for response, in this case, between very Happy and very Sad. The athlete’s image is licensed under CC by 4.0. Credits to Senior Airman Rusty Frank.

**Fig 3:**
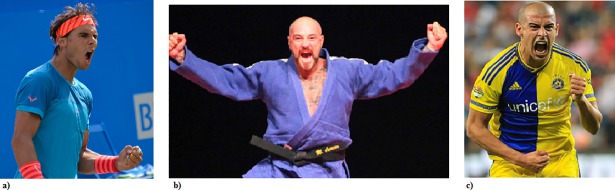
**(a) Dominant:** standing, hands clenched, head up, contracted upper and central face, mouth wide open and slightly funneled. **(b) Dominant/Happy:** Dominant features **plus** lip corners up and shoulders back. **(c) Dominant/Angry:** Dominant features **plus** lip corners down and asymmetrical body posture. Pictures are licensed under cc by 4.0. Credits go respectively to Carine06, Eva Rinaldi and Alon Luf.

A prototypical victory display is shown in Fig 1A, and prototypical defeat in Fig 1B. (1As the stimuli were selected three years ago, we were unable to get permission retroactively to show them, so we substitute them. In Figs 1, 2 and 3, we use images that are very similar to those used in the experiments, with the caveat that all our experiment stimuli showed full body displays. All the images are licensed under CC by 4.0 license).

We selected 49 pictures from the Cavicchio and Sandler study which portrayed athletes who had just won and displayed prototypical features of victory, 58 pictures of losing athletes that displayed prototypical features of defeat or loss, 22 pictures of athletes who had just won but displayed features of prototypical of victory and defeat together (‘mixed win’ pictures) and 14 pictures of athletes who had just lost and displayed mixed features (‘mixed loss’ pictures). We added 41 pictures of athletes with neutral displays, for a total of 184 pictures. The pictures were divided roughly equally by gender, and represented athletes from different parts of the world, photographed moments after they had won or lost a competition. Timing proximity to the moment of winning or losing was validated by YouTube searches for the videos of the sport events from which the pictures were taken. When the video was not available, timing of pictures at the moment of win or loss was validated through the sources' statements about pictures. The background of each picture was removed using photo editing software (Photoshop CS6 portable).

### Investigated emotions and experimental procedure

Participants were asked to rate the athletes’ emotional experience on a continuous visual analogue scale (VAS) from 0 to 100 (see Fig 2). They rated emotions that are commonly associated with victory and defeat in sports (1) Sad/Happy, (2) Ashamed/ Proud, (3) Disappointed/ Not Disappointed, (4) Angry/ Not Angry, (5) Frustrated/ Not Frustrated. These emotions were selected from the original 16 emotions in [45]. In [45], athletes reported their emotions before and after the match. In the current study, external observers were asked to report the perceived emotions of athletes that just won or lost a competition according to a subset of emotions from [44]. We selected Positive and Negative emotions that are expected to be associated with victory or defeat. Proud, Happy, and satisfied are typically associated with victory, while Angry, Sad, Ashamed, and Frustrated may be associated with defeat. In this study we did not include those emotions explored in [45] that evaluate the interaction between athletes and other people or objects, as we believe that these relationships might not be entirely clear to external observers.

Following [45], we explore the athletes’ arousal level from high arousal emotions such as Happy, Angry, Disappointed, Frustrated and Proud. Finally, we also asked participants to rate each picture for (6) Submissive/Dominant, with the understanding that this affective dimension is of critical importance in competitive interaction of humans as well as of other primates [46, 47] and would reflect the effect of the sports competitions, with winning producing increases in Dominance and losing producing increases in submissiveness.

To keep participants attentive and to reduce fatigue levels during the task, the pictures were divided into four groups. Each participant saw a quarter of the total number of pictures, in randomized order. An equal number of pictures displayed prototypical win features, prototypical loss features, mixed win and loss features and neutral postures/facial expressions. As recognition of some emotions requires longer time exposure than for others [48], participants were allowed to perform the task at their own pace. All pictures were presented using E-Prime, version 2.0.

### Methods of data analysis

All analyses were run in R 3.01. To analyze the data, we used the function lmer in package lme4 [49]. Since each emotion was scored along a Visual Analogue Scale (VAS) with a finite interval between 0 and 100, we hypothesized that the data follows a Poisson distribution. A first mixed effect regression was run to investigate the effect of picture type (winning, losing or neutral), picture features (prototypical or mixed) and their interaction, on each emotion scale score (dependent variable). Our model used the maximal random effects justified by the experimental design [50]: random intercepts for subjects (participants) and items (pictures), and random slopes by subjects and by items for picture type and picture features. P-values were obtained by likelihood ratio tests of the full model with the effect in question (win/loss or picture features) against the model without the effect in question.

A second mixed effect model was run to test which face and body features predicted the highest and the lowest scores for each emotion. As a result, we had the predictors listed in S1 Table. The predictors had different numbers of levels (see S1 Table). To standardize the contrast, we chose a *simple coding* scheme (see [51]). It was designed to compare the mean of the dependent variable for a given level (e.g., for head position, head neutral is the reference level) to the overall mean of that level. In both models, to address multicollinearity, we checked for high correlations between the predictors, and removed the predictors that were highly correlated. Finally, a post hoc Tukey test showed which face and body feature differed significantly from each other for each emotion. The test was performed using package multcomp in R [52].

## Results

Participants consistently rated pictures of athletes who had just won with significantly different scores than athletes who had just lost. Pictures displaying prototypical features had significantly different scores from pictures with mixed features (Est = -1.23, S.E = 0.14, df = 2, pr (Chisq)<0.001) and from neutral pictures (Est = -2.3, S.E. = 0.43, df = 2, pr (Chisq)<0.001). Mixed and neutral pictures tended to be scored in the middle of each emotion scale. In S2 Table we report the lmer model comparison and their results. In the follow up analysis, we investigated which face and body features predicted the highest and the lowest scores for each emotion. Face features (e.g. upper face and lower face AUs) and body features (e.g. head position, shoulders and standing position) were found to significantly predict the different emotion scores. The significant differences are reported in S3 Table.

Following earlier studies [45, 53–57], we interpreted groups of emotions as Positive (Proud and Happy) or Negative (Ashamed, Sad, Disappointed, Angry and Frustrated). Our findings revealed that certain features of emotions (specifically, anger, frustration and disappointment) tend to be associated with a cluster of features, and we explain the clustering in terms of Goal Obstruction [58], a category explained below. Arousal is another factor indicated by our results. Arousal is defined by Russel and Mehrabian [59] and Mehrabian [60] as ranging from inactivity and relaxation at the lower end to alertness and body tension at the high end. Certain emotions were reliably associated with face features that we attributed to high arousal, discussed in more detail below: Happy, Angry, Disappointed, Frustrated and Proud. Finally, we consider the Dominance/submission dimension to be particularly crucial in agonistic contexts and therefore this dimension was investigated by directly asking participants to rate the level of Dominance or Submissiveness of each athlete. Dominance is related to influence on others and control of the surroundings, while Submissiveness is described as experiencing a lack of these attributes [56, 61]. The salience of these emotional states is reflected in the fact that the largest blocks of features were reliably associated with either Dominance or Submissiveness. In Table 1A), we summarize the face and body features significantly associated with each emotion, according to participants’ responses. The features above the heavy black line are face features, and those below the line are body features. Colored cells indicate that the corresponding feature is associated with the emotion named atop the relevant column. For example, all of the colored cells under the column called ‘Angry’ represent the features of face and body that characterized pictures judged to be ‘very Angry’, i.e. at the bottom of the anger scale, by participants. Blocks of features that tended to cluster together are highlighted by sharing the same color. The labels above the chart indicate our interpretation of these clusters, based on literature cited above and in the discussion.

**Table 1:** a) Body and Face features characterizing the tested emotions, b) post-hoc clusters of emotions.

a)	**t**								
	**Submissive**	**Ashamed**	**Sad**	**Disappointed**	**Frustrated**	**Angry**	**Happy**	**Proud**	**Dominant**
Lip Corners Up (12)									
Lip Corners Down (15)									
Lower Lip Depressor (16)									
Stretched Mouth (20)									
Lip Part (25)									
Relaxed Mouth (26)									
Neutral Upper Face/ Closed Eyes (43)									
Not Visible									
Inner Brow Raise (1)									
Funneler (22)									
Chin Raiser (17)									
Open Mouth (27)									
Contracted Nose (9/10+38)									
Contracted Upper Face (4+6/4+6+7)									
On Knees									
Hands on Face									
Head Down (54)									
Relaxed Hands									
Head Up (53)									
Standing									
Clenched Hands									
Shoulders/Torso forward									
Asymmetry									
Shoulders Back									
	**Submissive**	**Ashamed**	**Sad**	**Disappointed**	**Frustrated**	**Angry**	**Happy**	**Proud**	**Dominant**
b)									
	**Submissive**	**Dominant**	**Negative**	**Positive**	**High Arousal**	**Resignation**	**Goal Obstruction**		

Table 1A) presents the distribution of face and body features predicting specific emotional states in participants’ responses. The emotions and the affective states are then grouped with respect to the broader underlying emotional components suggested by the literature (see Table 1B). For example, Happy and Proud have Positive valence [21], whereas Goal Obstruction is suggested for Disappointed, Frustrated and Angry [58], as explained below.

None of the tested emotions is characterize by a fixed, unique combination of face and body features, as predicted by the holistic emotion theory. Instead, most of the face and body features are distributed across multiple emotions and emotion categories. The largest group of face and body features characterizes emotions that are considered Dominant or Submissive in the literature [62–65]. Interestingly, whereas most features characterize Dominant or Submissive emotions, other features and group of features contribute additional components to the final display of each emotion. We detail our findings on face and body features in the following section.

### Face features

#### Face features of Dominance and Submissiveness

The largest cluster of face features characterizes **Dominance**, and they all contribute to contraction of the central and upper face, and widely opened mouth:

- Upper face: narrowed and lowered brows and raised cheeks (AUs4+6)
- Central face: contracted and dilated nose (AUs9+38), and raised upper lip and dilated nose (AUs10+38)
- Lower face: wide open mouth (AU27) and raised chin (AU17)

A different cluster of facial expressions characterizes **Submissive** emotional states. It consists of a relatively inexpressive face with either slightly open mouth or slightly parted lips (AU26). The upper face can be either neutral or with eyes closed (AU43). In many Submissive displays, the face is not visible at all, because the head is down, the hands are covering the face, or both.

#### Smaller face clusters and individual features

Two Actions Units which stretch the mouth (AU20 and AU16, colored in red in Table 1) typically occur with all emotional states in our dataset except for Ashamed and Submissive. We suggest that this combination of AUs signals **High Arousal.** Following [59] and [45] we expect high arousal in Happy, Angry, Disappointed, Frustrated and Proud. We further observe that opening and stretching the mouth creates a more open oral cavity to augment loud vocalization associated with high arousal and suggest that athletes displaying these features may have been emitting such vocalizations. High levels of Sadness have been attributed to very distressed and even weeping athletes, and, in our study, pictures of athletes judged to be at the lowest of the ‘Sad’ scale also correlated with AUs 20 and 16. Ashamed and Submissive, on the other hand, were associated with relaxed or covered faces.

Other face features convey nuances of emotion.

1. Raised lip corners (AU12 –in yellow in Table 1) associated with three emotional states: Happy, Proud and Frustrated;
2. Lip corners drawn down (AU15 –in grey) found the Negative emotions: Angry, Disappointed, Frustrated and Sad;
3. Lip funneler (AU22 –orange color), lips protruded outward, characterizes only Dominant, distinguishing this state from all other emotional states.
4. Raised inner brows (AU 1—inner eyebrows raised independently of the outer brows, colored purple in Table 1), characterizes Submissive only, distinguishing it from all other emotional states, whether in the Submissiveness or the Dominance dimension.

### Body cues

We found a specific cluster of bodily signals to be associated with Dominant emotional states. These are: standing position, head raised (AU53) and clenched hands. A standing position characterizes all emotional states in the Dominant range (Happy, Proud, Dominant, and Angry), and raised head and clenched hands also predict all of these, with one exception: Angry.

A diminished body posture is strongly associated with emotional states in the Submissive range (highlighted purple), typically characterized by kneeling, head bent down and hands covering the face. Disappointed is associated with Submissive head and hand position, but is distinguished from these by body posture, which is upright and not kneeling.

We find finer distinction in the bodily signals as well. Smaller groups of body features divide emotions into three groups:

a) Collapsed upper body (shoulders/torso lean forward), characterizes Submissive, Ashamed, and Sad emotional states (green). As we explain in the discussion, these states form a cluster associated with resignation. b) Asymmetrical posture characterizes three other Negative emotional states—Disappointed, Frustrated, and Angry (blue). These are grouped as emotions related to Goal Obstruction (see Discussion). c) The shoulders back feature characterizes only Positive emotional states—Happy and Proud—distinguishing them from the other Dominant states. In yellow we group these body features with the Positive facial feature, lip corners up, characterizing the same emotions, Happy and Proud.

## Discussion

The broadest distinction, i.e., consistently conveyed by the biggest blocks of features, is between Dominance and Submissiveness. This division makes good sense in the context of high-stakes physical competitions. The Dominance/Submissiveness dimension is orthogonal to a range of other emotional states. In the following, we discuss which features of facial expressions and body postures add elements of emotional meaning to the interpretation of complex displays, supporting the compositionality hypothesis.

### Dominance

Many face and body features characterize the Dominance display, indicating a good deal of redundancy. In a real-life confrontation, such redundancy would be very important, enabling other individuals to identify a potential threat quickly [66–68].

As for the face, Dominance is associated with contraction of the: cheek raise and lowering of the eyebrows, as well as nose wrinkle and upper lip raise. These facial actions mimic basic anatomical facial proportions which have been independently shown to be interpreted as dominant or masculine, since higher testosterone levels facilitate lateral growth of the cheekbones and forward growth of the eyebrow ridges [67, 68]. The contraction of the central part of the face enhances the facial width-to-height ratio, so that the face appears wider than it is in neutral position. Wide, highly masculine faces are considered more Dominant [69], and upper and central face contraction in our study may cause observers to associate facial expressions that mimic this phenomenon with Dominance. Interestingly, the Dominance display was the same across genders in the athlete pictures of our study.

A protruding chin (AU17) is characteristic of dimorphism between the sexes as well, and our study shows that raised chin is associated with expressions of Anger, Happiness, Pride, and Dominant–all emotional states belonging to the Dominance dimension and has also been shown to signal coping potentials and power [68]. AU 17 is achieved by contraction of the mentalis muscle which, raises the chin and the lower lip, concomitantly causing lip protrusion. This chin and lip configuration elongates the vocal tract, causing a lowering of the fundamental frequency of vocal cord vibration and other resonances in the vocal tract related to voice pitch [70, 71]. This low pitch effect is enhanced by funneling the lips (AU22), which associates significantly with Dominance. As Ohala [72] and many others have pointed out, deeper voices are linked to higher degrees of Dominance in many species. Even though participants saw only pictures without sound, experience might have led them to associate Dominance with these visual signals.

Body features corresponding to the Dominance dimension are typically these: standing posture, head tilted up, and clenched hands. This posture is found in all High Dominance emotions, except Angry, which is characterized significantly by standing posture (as well as asymmetry, discussed below), but not by head up and clenched hands.

### Submissiveness

In contrast with the intense facial actions in Dominance, the face cues of Submissiveness are generally very lax. They include slightly open, relaxed mouth, neutral upper face, closed eyes, or, alternatively, non-visibility of the face (because it is covered by the hands or the head is lowered). The general relaxation of facial muscles is an indication of surrender and resignation. Moreover, submissive athletes literally lose face, as they tend to cover their faces with the hands. The body signals of Submissiveness diminish the size of the body.

### Valence: The Positive—Negative dimension

Positive emotional states (Happy and Proud) were consistently predicted by lip corners up, shoulders drawn backward, and raised head posture. The facial expression of raised lip corners has been attributed to Positive emotions in numerous previous studies [4, 9, 73], including in congenitally blind athletes [74]. In both Happy and Proud, raised lip corners are accompanied by raised cheeks. The combination of these two Action Units is fundamental when expressing a “true” Duchenne smile [75, 76]. While Frustrated is also characterized by lip corners up, this action is not significantly associated with raised cheeks, which implies that athletes’ expressions judged to convey frustration often display “fake smiles” [77], which can communicate appeasement [78–80]. This interpretation is lent credence by the fact that Frustrated, unlike Happy and Proud, was also predicted by the more expected feature of lip corners down (though obviously not in the same pictures). The opposition we found between lip corners up for Positive emotions and lip corners down for Negative emotions is reminiscent of Darwin’s antithesis principle [81] regarding opposing physical expressions of opposite emotions.

For the body, shoulders shifted back is systematically associated with the Positive side of the scale. This posture may be less threatening than neutral or raised shoulders [82], or asymmetrical shoulders, discussed below. It is conceivable that the position of the shoulders could distinguish threatening from non-threatening postures within the broader dimension of Dominance.

### High arousal

Two action units affecting the mouth–mouth stretched sideways and depressed lower lip–appear to characterize high arousal in most of the affective states in this study. This result stems from the very nature of the present research which investigates the expressions of highly intense emotions in a competitive context, and the facial components responsible for this effect systematically reflect that. Only Submissive and Ashamed are not characterized by this mouth configuration according to our findings. Krumhuber and Scherer [83] also attribute these stretched mouth facial actions to high arousal emotions, with one difference: in their study these facial displays are mostly associated with highly aroused **Negative** emotions, while in our data, they occur with different emotional states regardless of their Valence, failing to be correlated with two of the Negative states, Submissive and Ashamed. We suggest that this distributional difference might be an artifact resulting from crucial differences in the stimuli used in the study and their context.

### Goal Obstruction and Resignation, two Negative responses with bodily cues

Two features of body position distinguish two types of emotional responses in Negative displays: Goal Obstruction and Resignation. The features are body asymmetry and collapsed upper body, respectively.

The feature of body asymmetry is found in specific Negative emotional states, and crosses the divide between Dominant and Submissive emotions. The feature relates to asymmetry of head or torso position, e.g., head tilted to the side, and one shoulder or side of the torso raised or pushed forward or up in relation to the rest of the body. Its appearance in pictures correlated significantly with participants’ judgments of the emotions, Angry, Disappointed and Frustrated. Ortony, Clore and Collins [58] propose that precisely these emotions are related to Goal Obstruction. According to participant judgments, then, asymmetrical body posture groups together three emotions that are all related to Goal Obstruction.

As noted, different emotional states are characterized by the feature, collapsed upper body (shoulders/body forward) according to participant judgments. There was a significant correlation between the emotional states, Submissiveness, Shame, and Sadness and the forward/collapsed upper body feature. Again, according to Ortony et al. [58], these emotions have something in common: evaluative disapproval and focusing on self, and we interpret these inward-looking emotions as associated with Resignation. So, whereas asymmetrical emotional states are reactions to Goal Obstruction, the collapsed states signal Resignation. That is, we propose that two emotional states with Negative valence are distinguished by asymmetrical vs. collapsed body features.

### Mapping emotional composition to the body

The experiment showed that complex emotional displays are comprised of features which are associated with emotional states, contributing to the overall interpretation of the display. For example, a display can be judged Dominant, and by adding raised lip corners and shoulders back, it is Happy (ecstatic) Dominance. Frustrated is distinguished from Sad mainly by the position of the body–asymmetrical for Frustrated and torso forward for Sad, and so forth.

In real life, intense emotional displays can be extremely complex, reflecting several emotional responses at the same time. These responses depend on the personality of the individual, expectations, the importance and relevance of the event prompting the display, and other factors [84]. For this reason, victory and defeat displays, even if they share the context of high stakes competitions, can each vary considerably.

Through experiments like the one we report here, we find generalizations across interpretations of participants that allow us to distill from a display those features which transmit a meaning related to an emotion or dimension.

In addition to supporting a compositional view of the expression of emotions, our results can also address some of the puzzling findings of previous studies. For example, Aviezer et al [29] (as reviewed in the Introduction) inferred from their results that facial features are not as reliable as body postures in participant judgements of athletes’ displays. Crucially, the researchers assumed a link between emotional Valence and what they term situational Valence. For them, winning a point in a tennis match has Positive situational valence, and should be associated with Positive emotions, while losing a point has Negative situational valence, and should be associated with Negative emotions. Participants were not able to judge Valence accurately from faces with bodies or just faces [29], and the authors reasoned that facial expressions are ambiguous. Even when participants were explicitly told that winners usually displayed an open mouth, participants still failed at differentiating Positive from Negative faces.

Our study takes a very different approach. We assumed that winning and losing athletes experience a variety of complex emotions, and our results bear this out. We find that large blocks of features cluster together with judgements of Dominance and Submissiveness, respectively, and understand from this result that Dominance and Submission (rather than Valence) are the most important dimensions in this context. Within these broad categories, features of facial expression, are not ambiguous, but rather, significantly correlate with nuances of emotion meaning. For example, a Dominant display can have Positive valence (Happy, Proud) with lip corners up, and Negative valence (Angry) with lip corners down (see Fig 3). These clusters of features are Dominant according to participants’ judgements, but some are likely to be associated with victory and some with defeat. These findings suggest that a finer measure than Positive/Negative valence is needed to understand the displays, and that the overriding contrast in this context is between Dominance and Submissiveness rather than Positive and Negative valence. In addition, the detailed results of our study show that testing for judgements about a wide range of emotions provides novel insight into the complexity of the expression and interpretation of emotion.

### Limitations of the current study

Participants rated each emotion along several emotion and affective scales, as described in the method section. However, some concerns arose regarding the anchoring in the case of the continua Happy/Sad and Ashamed/Proud, as these emotions were at the two sides of the respective continuum. This was true, however, only for the abovementioned emotions as they are antonyms. For the other emotions (e.g. Angry) the rating scale ranged from 0, not Angry at all, to 100, high level of anger. We suggest that in future studies it may be worth using a separate rating scale for each individual emotion, including the opposite ones such as Happy/Sad.

Another possible limitation of the current study is participants’ gender imbalance. It has been suggested that females perceive and express emotions in different ways than men. However, recent studies found that those differences are very small [85] or not significant at all [38, 39]. Finally, we expect our results to be highly context dependent. As we examined only emotions in the context of victory and defeat, our results do not make predictions about emotions in everyday interactions.

## Conclusions and directions for future research

Overall, we find that different components of displays of intense emotion by athletes are reliably matched to emotional dimensions or states. First, our findings regarding the primacy and salience of Dominance and Submission are sound from an evolutionary point of view. The two dimensions are characterized by complex configurations of features—open body posture and enhanced facial features that signal Dominance, and the prostrated posture with occluded or diminished face that signal Submissiveness. These highly salient, complex displays clearly communicate power or surrender to others in the context of high-stakes, physical confrontation. Additional components of emotional displays add other ecologically relevant distinctions, resulting in diverse emotional interpretations, such as intense Pride, Anger, or Resignation.

Regarding the main point of our investigation, our findings give credence to a compositional model of emotion expression by the body, in which complex displays are built up of features that contribute their emotional meanings to the whole, and which distinguish the interpretation of one complex from that of another.

In terms of compositionality, this line of research demonstrates a nontrivial property in common with language. In both cases, we see that humans display and interpret complex communicative displays in terms of their component parts. However, there are also instructive differences. First, we believe that the emotional signals are far more general and much more context-dependent than the units of language. This implies that their manifestations, combinations, and interpretations may differ widely in different contexts. In fact, a crucial difference between emotional displays and language is the role of context. We expect that the same features in emotional displays can be interpreted in quite different ways depending on the context. While context is important for language as well, even complex compositional messages can be understood with no context (“out of the blue”), and, though context can make a difference in interpretation of language too, we expect that there is much less reliance on context in language compared to emotional displays. This is one of the strengths of language, and the comparison deserves to be tested rigorously.

Furthermore, the combination and recombination of features are governed by the nature and composition of human emotion, reflecting responses to our ecological environment, and constrained by it. In contrast, language allows us to imagine and communicate thoughts and ideas that are not of the here and now and is constrained by rules that are quite specific to the domain and allow for the expression of an infinite number of utterances about anything we can conceive of.

If linguistic and emotional expressions share the key feature of compositionality, the door is now open to the investigation of other similarities and differences between these two communicative domains. Some similarities and differences between the domains have been revealed here, paving the way, we hope, for future research that will explore and rigorously compare the two.

Our analysis makes additional specific predictions for future research. The associations that we describe between face/body features and emotional states and dimensions are derived from participant responses to many pictures of spontaneous displays of emotion. In future research, we intend to use embodied agents to test the prediction that the features in question distinguish emotional states by manipulating targeted features and groups of features (see Sandler [35] for examples of embodied agents displaying intense emotions).

More than one feature usually correlates with an emotional state in judgements of photographs of real athletes, e.g, lip corners up as well as shoulders back for Happy displays. Since our results regards natural stimuli with complex displays, we are unable to tell if these features contribute information independently. For that, future research will use embodied agents whose expressions and postures can be selectively manipulated. For example, we will present a Dominant face and body posture, changing only one feature (e.g, body asymmetry). We expect participants to judge the character displaying Dominant face and body features plus body asymmetry as Angrier than the same character with symmetrical body. Adding lip corners down to a Dominant posture will also determine a shift of judgments from Dominant to Angry. Adding both lip corners down and asymmetry to a Dominant display will lead to the highest scores/faster recognition of Anger. Mixed displays (e.g, kneeling posture with head up and lip corners up) will allow us to test the relative contribution of different face and body features. Finally, as noted, we expect that the expression of emotion is highly context dependent. Another direction for future research is to examine other contexts, perhaps less extreme, using similar methodology and coding categories to those laid out here, with the aim of fully exploring the effect of context on the interpretation of emotion and its components. All in all, we hope that our results open the door to clear new directions of research into the compositionality of human expression.

## Supporting information

S1 Table List of the model predictors and corresponding number of levels of each predictor. (DOCX)

S2 Table p values of the lmer model. The p values were calculated by likelihood ratio tests, comparing the full lmer model against the model without the effect. Tests were conducted using the function anova in package lmer in R.
(DOCX)

S3 Table Face and body features predicting the highest and lowest scores for each emotion tested. The analysis was conducted using package multcomp in R.
(DOCX)
