# Supplementary material for: Compositionality in the language of emotion
Source: PLoS One. 2018 Aug 15;13(8):e0201970. doi: 10.1371/journal.pone.0201970 (PMC6093664; doi:10.1371/journal.pone.0201970)
Supplement: S1 Table — (DOCX) [file pone.0201970.s001.docx]

Table 1: List of the model predictors and corresponding number of levels of each predictor.

| Face | | |
| --- | --- | --- |
|  | Number of levels | Reference level |
| Upper Face | 9 | Neutral |
| Nose Area | 4 | Neutral |
| Lower Face | 13 | Neutral |
| Body | | |
|  | Number of levels | Reference level |
| Head position | 5 | Neutral |
| Hands position with respect to face/head | 5 | Along the body |
| Shoulder position | 7 | Neutral |
| Torso position | 7 | Straight torso |
| Standing | 7 | On feet |
| Handshape | 9 | Neutral/relaxed |
