# Supplementary material for: Compositionality in the language of emotion
Source: PLoS One. 2018 Aug 15;13(8):e0201970. doi: 10.1371/journal.pone.0201970 (PMC6093664; doi:10.1371/journal.pone.0201970)
Supplement: S2 Table — The p values were calculated by likelihood ratio tests, comparing the full lmer model against the model without the effect. Tests were conducted using the function anova in package lmer in R. (DOCX) [file pone.0201970.s002.docx]

| **WL= win/loss**  **PT=prototypical vs mixed vs neutral** | **Win vs loss** | **Prototypical vs mixed** | **Prototypical vs neutral** | **Mixed vs neutral** |
| --- | --- | --- | --- | --- |
| **Ashamed/Proud**      **fit<-**  **glmer (AshamedProudScore ~WL+PT+0+PT\| Subject) +(1\|Item),**  **family="poisson", data=d,**  **ML=T)** | Pr(chisq)< 0.001    **fit2<-**  **glmer (AshamedProudSc ore~PT+0+PT\| Subject )+**  **(1\|Item),**  **family="poisson", data= d, ML=T)**    **anova (fit, fit1)** | Pr(chisq)< 0.001    **fit3<-**  **glmer (AshamedProudScore~ WL+ 1\|Item),**  **family="poisson", data=d,**  **ML=T)**      **Anova (fit, fit3)** | Pr(chisq) <  0.02    **Summary (glht (fit, mcp(PT="Tukey")))** | Pr(chisq) <  0.01    **Summary ( glht(fit, mcp(PT="Tukey")))** |
| **Sad/Happy** | Pr(chisq)< 0.01 | Pr(chisq)< 0.01 | Pr(chisq)<0.001 | Pr(chisq)< 0.01 |
| **Frustrated** | Pr(chisq)< 0.01 | Pr(chisq)< 0.001 | Pr(chisq)< 0.03 | Pr(chisq)< 0.001 |
| **Disappointed** | Pr(chisq)< 0.01 | Pr(chisq)< 0.0001 | Pr(chisq)< 0.001 | Pr(chisq)< 0.04 |
| **Angry** | Pr(chisq)< 0.001 | Pr(chisq)< 0.01 | Pr(chisq)< 0.01 | Pr(chisq)< 0.001 |
| **Submissive/Dominant** | Pr(chisq)< 0.001 | Pr(chisq)< 0.01 | Pr(chisq)< 0.01 | Pr(chisq)< 0.01 |

**Table 2: p values of the lmer model. The p values were calculated by likelihood ratio tests, comparing the full lmer model against the model without the effect. Tests were conducted using the function anova in package lmer in R.**
