# Supplementary material for: Compositionality in the language of emotion
Source: PLoS One. 2018 Aug 15;13(8):e0201970. doi: 10.1371/journal.pone.0201970 (PMC6093664; doi:10.1371/journal.pone.0201970)
Supplement: S3 Table — The analysis was conducted using package multcomp in R. (DOCX) [file pone.0201970.s003.docx]

Table 3: Face and body features predicting the highest and lowest scores for each emotion tested. The analysis was conducted using package multcomp in R.

|  | Ashamed/ Proud | VerySad/Very Happy | Frustrated / NotFrustrated | Disappointed/ notDisappoint ed | Angry/not Angry |  | Submissive/ Dominant |
| --- | --- | --- | --- | --- | --- | --- | --- |
| Face |  |  |  |  |  |  |  |
| AU12  (lip corner up) | Est=-0.82 p<0.01 | Est=-1.5 p<0.01 | Est=-0.6 p<0.04 |  |  |  |  |
| AU15  (lip corner down) |  | Est=-1.5 p<0.001 | Est=-0.8 p<0.01 | Est=-1.5 p<0.001 | Est=-1.2 p<0.001 |  |  |
| AU16  (lower lip depressor) | Est=-0.82 p<0.01 | Est=-1.5 p<0.001 |  | Est=-1.5 p<0.001 | Est=-1.2 p<0.001 |  | Est=-1  p<0.001 |
| AU17  (Chin raiser) | Est=-0.82 p<0.01 | Est=-1.5 p<0.001 |  |  | Est=-1.2 p<0.001 |  | Est=-1 p<0.001 |
| AU22  (Funneler) |  |  |  |  |  |  | Est=-0-86 p<0.001 |
| AU20  (Stretched Mouth) | Est=-0.82 p<0.01 | Est=-1.5 p<0.01 |  |  | Est=-0.6 p<0.001 |  | Est=-1  p<0.001 |
| AU25  (Lip part) | Est=-0.82 p<0.02 | Est=-0.9 p<0.02 | Est=-1.05 p<0.001 | Est=-1 p<0.001 |  |  | Est=-1 p<0.001 |
| AU26  (jaw dropped) |  | Est=-1.6 p<0.001 | Est=-0.6 p<0.04 | Est=-1.34 p<0.001 |  |  | Est=-1  p<0.001 |
| AU27  (mouth stretch) | Est=-0.82 p<0.001 | Est=-1.5 p<0.001 |  |  | Est=-0.6 p<0.001 |  | Est=-0.49 p<0.001 vs neutral -0.27  (not visible) |
| Aus 9+38/10+38  (contracted nose area) | Est=-0.38 p<0.001 | Est=-0.5 p<0.001 |  |  | Est=-0.5 p<0.001 |  |  |
| Neutral Nose Area | Est=-0.38 p<0.001 | Est=-0.5 p<0.001 |  |  |  |  |  |
| AUs 1+2 eyebrow raised) |  |  |  |  |  |  | Est=0.8 p<0.01 |
| AUs 4+6/  4+6+7  (contracted upper face) | Est=-1.25 p<0.001 | Est=-0.8 p<0.001 |  |  | Est=-0.5 p<0.005 |  | Est=0.8 p<0.01 Est=0.43 p<0.01 (4+6+7) -0.87 p<0.001  (4+6+7) |
| Neutral upper face/ closed eyes | Est=-1.25 p<0.01 | Est=-0.8 p<0.01 | Est=-1.2 p<0.04 | Est=-1.21 p<0.001 |  |  | Est=0.43 p<0.001 |
| Face not Visible | Est=-8.27 p<0.01 | Est=-1.2 -0.61 p<0.01 |  |  |  |  | Est=-0.87 p<0.001 -0.49  P<0.01  (vs 4+6) |
|  | Ashamed/ Proud | Very  Sad/Very  Happy | Frustrated /  Not  Frustrated | Disappointed/ not  Disappointed | Angry/not Angry |  | Submissive/ Dominant |

|  | Ashamed/ Proud | Very  Sad/Very  Happy | Frustrated /  Not  Frustrated | Disappointed/ not  Disappointed | Angry/not Angry |  | Submissive/ Dominant |
| --- | --- | --- | --- | --- | --- | --- | --- |
| Body |  |  |  |  |  |  |  |
| Head Down | Est=-0.79 p<0.01 | Est=-0.36 p<0.001 | Est=-0.27 p<0.01 | Est=-0.56 p<0.01 | n.s. |  | Est=-0.6 p<0.01 |
| Head Up | Est=-0.79 p<0.01 | Est=-0.36 p<0.001 | Est=-0.27  p<0.01 | Est=-0.56  p<0.01 | n.s. |  | Est=-0.6 p<0.01 |
| Hands on Face | Est=-0.48  p<0.001 | Est=-0.35  p<0.01 | Est=-0.44  p<0.01 | Est=-0.5  p<0.001 | n.s. |  | Est=-0.75  p<0.001 |
| Hands away from face/body | Est=-0.48 p<0.001 | Est=-0.35 p<0.01 | Est=-0.44 p<0.01 | Est=-0.5 p<0.001 | n.s. |  | Est=-0.75 p<0.001 |
| Shoulders forward | Est=-0.38  p<0.01 | Est=-0.52  p<0.001 | n.s | n.s | n.s. |  | n.s |
| Shoulders Back | Est=-0.38 p<0.01 | Est=-0.52 p<0.001 | n.s | n.s | n.s. |  | n.s. |
| Asymmetrical shoulders | n.s. | n.s. | Est=-0.65  p<0.001 | Est=-0.45  p<0.001 | Est=-0.4  p<0.01 |  | n.s |
| Torso back | n.s. | n.s. | n.s. | n.s. | n.s. |  | Est=-0.55  p<0.01 |
| Torso forward | n.s. | n.s. | n.s. | n.s. | n.s. |  | Est=-0.55  p<0.01 |
| Standing | Est=-0.6  p<0.001 | Est=-0.65  p<0.01 | Est=-0.2  p<0.001 | Est=-0.58  p<0.001 | Est=-0.32  p<0.001 |  | Est=-0.61  p<0.001 |
| On knees/lying | Est=-0.6  p<0.001 | Est=-0.65  p<0.01 | Est=-0.2  p<0.001 | Est=-0.58  p<0.001 | Est=-0.32  p<0.001 |  | Est=-0.61  p<0.001 |
| Clenched hands | Est=-0.28  p<0.001 | n.s. | n.s. | n.s. | n.s. |  | Est=-0.65  p<0.01 |
| Relaxed/neutral hands | Est=-0.28 p<0.001 | n.s. | n.s. | n.s. | n.s. |  | Est=-0.65 p<0.01 |
|  | Ashamed/ Proud | Very  Sad/Very  Happy | Frustrated /  Not  Frustrated | Disappointed/ not  Disappointed | Angry/not Angry |  | Submissive/ Dominant |
